# Airway trauma: a review on epidemiology, mechanisms of injury, diagnosis and treatment

**DOI:** 10.1186/1749-8090-9-117

**Published:** 2014-06-30

**Authors:** Christos Prokakis, Efstratios N Koletsis, Panagiotis Dedeilias, Fotini Fligou, Kriton Filos, Dimitrios Dougenis

**Affiliations:** 1 Department of Cardiothoracic Surgery, University of Patras, School of Medicine, Patras, Greece; 2 Department of Cardiothoracic Surgery, Evaggelismos Hospital, Athens, Greece; 3 Department of Anesthesiology and Intensive Care, University of Patras, School of Medicine, Patras, Greece

**Keywords:** Trachea, Injury, Airway, Bronchoscopy, Primary repair, Surgery, Conservative, Delayed presentation

## Abstract

Airway injuries are life threatening conditions. A very little number of patients suffering air injuries are transferred live at the hospital. The diagnosis requires a high index of suspicion based on the presence of non-specific for these injuries symptoms and signs and a thorough knowledge of the mechanisms of injury. Bronchoscopy and chest computed tomography with MPR and 3D reconstruction of the airway represent the procedures of choice for the definitive diagnosis. Endotracheal intubation under bronchoscopic guidance is the key point to gain airway control and appropriate ventilation. Primary repair with direct suture or resection and an end to end anastomosis is the treatment of choice for patients suffering from tracheobronchial injuries (TBI). The surgical approach to the injured airway depends on its location. Selected patients, mainly with iatrogenic injuries, can be treated conservatively as long as the injury is small (<2 cm), a secure and patent airway and adequate ventilation are achieved, and there are no signs of sepsis. Patients with delayed presentation airway injuries should be referred for surgical treatment. Intraoperative evaluation of the viability of the lung parenchyma beyond the site of stenosis/obstruction is mandatory to avoid unnecessary lung resection.

## Introduction

Airway trauma is a life threatening condition which may be a result of blunt and penetrating injuries to the neck and chest, as well as medical procedures that may injure the airway. The presence of concomitant severe injuries and symptoms and signs which are no specific for this type of injury may delay the diagnosis and lead to early fatal outcome (ventilator failure, asphyxiation from airway obstruction, or death from tension pneumothorax) or late sequela such as airway stenosis and recurrent pulmonary infections [1]. Therefore, prompt diagnosis is mandatory for the survival of these patients. The treatment of these patients is similarly challenging: it includes, predominantly a secure and patent airway which will allow adequate ventilation and then the repair of the injury with a smaller impact on the respiratory function and the quality of life of the patients [2].

The aim of this paper is to review the epidemiology, the mechanisms of injury, the diagnosis and the management of the patients suffering from airway injuries.

## Review

### Epidemiology

The true incidence of TBI is still unknown as 30–80% of these trauma victims still die at the scene of the accident [3]. Currently, the incidence of TBI among trauma patients with chest and neck injuries, including those that died immediately, is estimated at 0.5-2% [4]. An anticipated incidence of 3-6% for cervical trachea injuries should be assigned to penetrating neck injuries, whereas the incidence of airway injury in patients with penetrating chest trauma ranges from <1% to 2% [5,6]. In a relatively recent report by Kummer et al. [7] the incidence of traumatic airway injury was 0.4% and 4.5% for blunt and penetrating traumas respectively. In a large autoptic study of 1187 trauma patients Bertelsen and Howitz [8] found 33 patients (0.03%) who had TBI; 27 of them died immediately after the accident and 24 had severe associated injuries. However, this is a unique case since most autoptic studies have revealed airway injuries in 2.5-3.2% of the victims [3]. The incidence of iatrogenic TBIs may be 1 event every 20.000-75.000 elective orotracheal intubations, increasing up to 15% when the procedure is emergently performed, and 0.2-0.7% for percutaneous dilatative tracheotomies [9,10]. In a relatively recent review Minambres et al. [11] reported, estimated incidence of tracheal rupture after endotracheal intubation, to be among 0.05% to 0.37% in the last decade.

The mortality from traumatic TBI’s has decreased from 36% before 1950 and 30% in 1966 to 9% in 2001 [3,12,13]. Finally, it is estimated that major co-existing injuries can be found in 40-100% of the trauma victims with TBI, and may have a significant impact on the final outcome of these patients [5,14]. In penetrating neck injuries traumatizing the airway, rupture of the esophagus is the most common accompanying injury occurring in up to 43% of the patients [4].

### Mechanisms of injury

The cervical trachea is at high risk for injury after penetrating trauma to the neck. However, airway injuries at the neck may also result from blunt trauma. Neck hyperextension can result in tracheal tears, paramedian vertical fractures of the larynx and trachea, and/ or even lead to complete laryngo-tracheal separation [2,15]. On the contrary direct blows usually injure the thyroid and cricoids cartilages [16]. A specific type of injury combining both mechanisms is the one produced when neck hyperextension from sudden deceleration during a vehicle accident is followed by direct collision of the neck to the steering weal or the dashboard, the so called “padded dashboard syndrome” [15,17]. The trachea can also be injured when it’s violently compressed, along with the esophagus, against the cervical spine [5]. Finally, the trachea can be traumatized in front-on collision by the seat belt, and sometimes, by the sudden increase of intratracheal pressure against a closed glottis caused by the improper use of the seat belt [18].

In blunt chest traumas Kisser et al. [12] proposed three mechanisms for TBI’s. The first is the “explosive rupture” where, after the chest is crushed, there is a quick rise of the pressure in the airway while the glottis is reflexively closed. Once the pressure exceeds the elasticity of the tissues they explode. The second mechanism involves the development of shearing forces at fixed points of the airway (cricoid cartilage and carina) due to its movement during sudden deceleration. The third one occurs when the chest is compressed along its anterior-posterior axis. As the lungs remain fixed to the chest wall because of the negative pressure existing between them and the parietal pleura, and the compression pulls them apart, excessive tensile forces may lacerate the airway at the level of the carina. In most cases the airway injury results from the combination of these three mechanisms. The first mechanism mostly occurs in lacerations of the posterior wall, while the other two, and especially the last one, can clearly explain why 75-80%t of the injuries occur within 2.5 cm from the carina [2,3,19].

### Clinical and radiological studies

The symptoms and signs of TBI depend on the site and the severity of the injury and most of them are not specific for this kind of injury. Subcutaneous emphysema is the most common finding in TBI occurring in up to 87% of the patients [20]. Laceration of the mediastinal pleura and/or bronchial injuries may allow air to enter in the pleural cavity; pneumothorax occurs in 17-70% of the patients with TBI’s [21-23]. A massive air leak and the inability to re-expand the lung after tube thoracostomy are highly indicative of a TBI [24]. Attempts to apply negative pressure to the chest tube may increase the air leak and deteriorate the respiratory function of the patient [2,23]. The air can also be trapped in the mediastinum and a crackling rumor concurrent with the heartbeat may be heard on the auscultation over the precordium (Hamman’s sign) [25]. Dyspnea, tachypnea and respiratory distress are found in 59-100% of the patients [19,23], while haemoptysis can be seen in up to 74% of the cases [24]. Voice changes varying from hoarseness to aphonia may result from laryngeal fractures, laryngo-tracheal separation, vocal cord tears and recurrent laryngeal nerve injury [26]. Finally, air escape from a penetrating neck trauma should be considered diagnostic for airway injury [19,27].

The most common findings in chest x-rays are subcutaneous emphysema, pneumomediastinum and pneumothorax. If subcutaneous emphysema exists and the hyoid bone appears high in the neck on lateral radiographics of the cervical spine there is high possibility for transection of the cervical trachea [28]. Other signs that may become apparent include: tracheal deformity, a defect in the tracheal contour, an endotracheal tube out of place and the tube’s cuff over-inflated and protruding beyond the edge of the tracheal wall [14]. When the bronchus is circumferentially transected and detached the lung may collapse towards the diaphragm and posteriorly, below its hilar attachment, and not inwards like every other case of pneumothorax; this is the sign of the fallen lung and although rare it is diagnostic for bronchial injury [29]. It is believed that up to 10-20% of the patients with TBI may have no signs of TBI on chest x rays [13].

Chest computed tomography (CT) can readily detect all the aforementioned signs for airway trauma. Although highly diagnostic for laryngeal trauma it had been considered less specific for the diagnosis of TBI. However, Chen et al. [30] reported that CT imaging helped the authors to diagnose the airway injury in 10/14 patients (71%). Relatively recent evidences indicate that multi-slice detector CT imaging with MPR/3D reconstruction of the images can significantly increase the diagnostic accuracy of the procedure up to 94-100% [31,32]. Virtual bronshoscopy is a novel technique which comprises a computer generated volumetric reconstruction of the tracheobronchial tree based on MPR/3D images obtained during multi-slice CT imaging of the airway [33]. The technique simulates the findings at conventional bronshoscopy, and therefore it could represent a valid alternative for the evaluation of the airway, even for the detection of TBI in selected patients with more stable airway injuries [34,35].

### Airway management and bronchoscopy

Although rapid sequence endotracheal intubation is frequent in trauma patients in those with airway injury may result disastrous. The pressure over a fractured cricoid may dislocate it enough to completely distort the upper airway, change the view of the physician performing the intubation or even lead to complete airway transection and obstruction [16]. Attempts to blindly overpass an upper airway injury may worsen the laceration and/or create false passage of the tube [36]. Finally, intravenous induction and neuromuscular blockade should be avoided since apnea and the loss of the smooth muscle tone may lead to complete collapse of an already traumatized and distorted airway kept functional by the surrounding musculature [37,38]. Therefore, spontaneous breathing of the patient should be preferred until safe airway has been achieved.

As bronchoscopy represents the procedure of choice to locate the site of the rupture, its extension and depth, and to make sure that the tube’s cuff is inflated beyond the site of the injury, endobronchial intubation over a flexible bronchoscope is the preferred method for airway management and for the definitive diagnosis of a TBI [4,5,39]. Common findings of TBI include tearing of the wall, blood in the airway and collapsed airway with inability to view and assess the part of the tracheobronchial tree distally to the site of the injury [3,19]. Sometimes the view may be impaired by the presence of blood clots and tissue debris, and if critical airway stenosis exists the bronchoscope may itself precipitate airway obstruction [16,40]. Rapidly desaturating and/or hemodynamically unstable patients may not cooperate to perform the procedure without general anesthesia. In these scenarios rigid bronchoscopy as the patient receives inhalation induction, while maintaining spontaneous ventilation, may allow evacuation of blood and debris, good examination of the airway, bridging of an airway defect, and good ventilation in an already anesthetized patient [40,41]. The main limitation for rigid bronshoscopy is the need to extend the neck to the shoulder, which cannot be performed if cervical spine injury is suspected and/or has not been ruled out [13,16].

Injuries to the carina and main bronchi represent a challenging setting for airway management. Blind endotracheal intubation with the tube’s cuff inflated proximally to the injury and positive pressure ventilation may increase the air leak and lead to severe respiratory insufficiency and pending respiratory arrest [42]. Rapid bronchoscopic evaluation of the airway should be undertaken and once the site of the injury has been identified single lung ventilation should begin; the tube can be guided into the uninjured bronchus in case of a bronchial rupture, or we may use a double lumen tube to intubate the patient [3]. For patients suffering from post-intubation TBI to the distal trachea it has been reported that it’s possible to place small low pressure cuffed tubes into both bronchi through the tracheostomy [43]. In extremis, urgent thoracotomy and placement of intrabronchial catheters in both bronchi through the chest wound may allow adequate ventilation in patients with distal airway injuries rapidly desaturating despite the standard airway management procedures [44].

Tracheostomy is not routinely performed in airway trauma. It should be done under local anesthesia in patients with significant laryngo-tracheal injuries with complete or pending airway obstruction and when endotracheal intubation is considered unwise and unsafe as in the presence of concomitant craniomaxillofacial injuries [16,45]. Moreover, it should be performed in every patient with TBI after failed attempts for endotracheal intubation [2,46]. In patients with penetrating cervical tracheal injuries the insertion of a tracheostomy tube through the wound is the best way to secure the airway and spares tissue for future repair if this is deemed necessary [41].

### Definitive management

#### Surgery

The rational for surgery can be summarized in: a. closing of the airway defect to improve ventilation, b. preventing mediastinal spillage and infection and c. avoiding spontaneous healing complications which can lead to airway stenosis and recurrent pulmonary infections.

Cervical trachea injuries are better accessed through a collar incision, while intrathoracic trachea injuries, above the level of the aortic arch, are better exposed by extending the transverse cervical incision into an upper sternotomy [19,23,47,48]. The collar incision can also be extended along the border of the sternocleidomastoid muscle to explore the carotid sheath when vascular injury is suspected [21,41]. Posterior tracheal wall lacerations can also be treated via left cervicotomy [49] or collar incision followed by longitudinal tracheotomy to gain access to the membranous wall [50,51]. Unilateral cervical incisions, especially the ones on the left, are suited for the management of combined tracheal and esophageal injuries [52]. The lower part of the intrathoracic trachea, the carina, the right bronchus and the proximal 2 cm of the left one are mainly approached via right thoracotomy at the 4^th^ intercostal space. The alternative includes median sternotomy followed by posterior transpericardial access to the airway once the ascending aorta and the superior vena cava have been mobilized [53]. Finally, abruptions of the left bronchus close to the bifurcation of the lobar bronchi are best treated via left thoracotomy [24].

Small tears and lacerations should be closed with direct sutures, while complete or partial transections require debridement of the infected and devitalized tissues, trimming of the edges of the injured airway and end to end anastomosis. Extensive circumferential mobilization of injury edges should be avoided because of the risk to devascularize the anastomosis and create healing problems with anastomotic dehiscence and/or later on airway stenosis and subsequent infection [2]. The repair should be done with absorbable sutures with the knots tied on the outside to avoid the development of granulomas that may erode into the lumen [23,54]. Protective tissue flaps (pericardium, muscle flaps, pleura, mediastinal fat) can be used to cover the sutures or the anastomosis and separate them from the esophagus in combined trachea-esophageal injuries [19,24,55]. When resection is necessary a tension free anastomosis can be performed by flexing the neck before the repair; the flexion should be maintained post-operatively by a chin to chest stitch until the anastomosis is healed. Other alternatives include laryngeal release maneuvers, division of the inferior pulmonary ligaments and pericardial release of the pulmonary hilum. Tracheostomy through the wound is an alternative to primary repair in penetrating neck traumas with an anterior tracheal injury less than half of the airway’s circumference and/or involving up to 2 cartilaginous rings [56]. If there is extensive tissue damage and a large defect precluding primary repair an end tracheostomy must be created; the proximal trachea is closed and the distal one is circumferentially sewed to the skin after fixing the trachea to the prevertebral fascia to avoid its retraction into the mediastinum [41]. An attempt for definitive repair can be performed later on. Finally, protective tracheostomy below the site of the repair should be done in laryngo-cricoid-tracheal injuries with suspected injury to both recurrent laryngeal nerves, when long term mechanical ventilation is anticipated and when the repair is considered at risk for complications. Bronchial injuries are treated by applying the same principles. However, extensive bronchial damage, co-existing pulmonary vascular injuries, and/or irreversible destruction of lung parenchyma may necessitate lung resection for the effective control of the injury [20,48]. Pneumonectomy in this setting is considered a highly risky procedure; therefore, every attempt should be made to minimize the loss of pulmonary functional tissue mainly by performing a bronchoplastic procedure [57-59] Although cardiopulmonary bypass is poorly tolerated by these patients because of the need for antigoagulation, selected rapidly desaturating and hemodynamically unstable patients with complex or combined airway and vascular injuries may benefit by the institution of cardiopulmonary bypass; the patient can be stabilized, the repair is done in better conditions and if hilar vascular injury co-exists the repair of the vessel can be feasible, thus avoiding the need for pneumonectomy [53,60-62].

When surgery is performed early after the injury the long term outcome is good for over 90% of the patients [5]. Table 1 summarizes both surgical techniques and outcome of patients undergoing surgery for airway injury. The 30 day mortality varies from 0% to 44.4% [39,47,49,63] and is primarily affected by the presence of concomitant injuries, the time between diagnosis and treatment and the need for pneumonectomy [12,57,64]. Although complications can be seen in up to 25.8% of the patients [48], major morbidity is mostly related to septic complications from long ICU recovery and anastomotic dehiscence [20,39,49,57]. Long term complications include airway stenosis [48], and phonation problems from injuries to the larynx and recurrent laryngeal nerves [65].

**Table 1:** Results of surgery in patients with traumatic airway injuries

**Study**	**No**	**Operative approach**	**Surgery**	**Mortality**	**Complications**
Barmada (23)	7	R Thoracotomy: 5	L. pneumonectomy: 1	0%	Wound infection: 2
		L Thoracotomy: 2	ML. lobectomy: 1		Sputum retention: 1
			Segmentectomy: 1		
			Primary suture: 4		
Rossbach (19)	32	Collar incision: 16	Primary suture: 23	6%	Pneumonia: 3
		Collar + sternotomy:1	Tracheostomy: 3		Suture granuloma:2
		Sternotomy: 5	Primary anastomosis: 2		Wound infection:1
		R Thoracotomy: 9	R pneumonectomy: 2		
		L Thoracotomy: 1	ML Bilobectomy: 1		
Cassada (39)	18	Collar Incision: 3	Primary suture: 13	5.6%	Sepsis: 2
		L cervicotomy: 5	Tracheostomy: 2		BPF: 1
		Sternotomy: 1	Repair + tracheostomy: 1		
		R thoracotomy: 8	L. pneumonectomy: 1		
		L thoracotomy: 1	Lobectomy: 1		
Mussi (47)	16	R thoracotomy: 5	Primary suture: 13	0%	Suture granuloma: 1
			Primary anastomosis: 2		
		Collar inciusion: 11	Thyroid cartilage suture: 1		
Hofmann (49)	18	R thoracotomy: 17	Primary suture: 18	44.4%	Suture dehiscence: 1
		L thoracotomy: 1			
Balci (48)	32	Thoracotomy: 19	Primary suture: 25	21.8%	Atelectasis/pneumonia: 2/2
		Sternotomy: 2	Primary anastomosis: 6		Prolonged air leak: 1
		Clamshell: 1	RUL lobectomy: 1		Bleeding: 1
		Neck incisions: 10			Empyema: 1
					Wound infection: 1
					Airway stenosis: 3
Richardson (57)	60	Cervical incision ± sternotomy: all laryngotracheals and most tracheal	Primary suture: 8 (2 laryngotracheal)	13.3%*	Failed repair: 1
			Primary anastomosis: 25		Phonation problems: 1
		Thoracotomy: 24 bronchial injuries and some lower tracheal	Bronchial reconstruction: 10		Airway stenosis: 5
			Pneumonectomy; 14		Empyema: 3 (BPF in 2)
					Suture granuloma: 6
			Delayed presentation: primary repair (1), resection and anastomosis (1), pneumonectomy (1)		Pulmonary hypertension/cor pulmonale: 3
					Respiratory insufficiency: 1
Gomez-Caro (70)	11	Cervical incision: 3	Primary suture: 8	0%	
		Thoracotomy: 8	Primary anastomosis: 1		
			Tracheostomy: 1		
			R. pneumonectomy: 1		
Koletsis (20)	22	Collar incision ± partial sternotomy: 11	Primary suture: 19	4.6%	Airway stenosis: 1
		Thoracotomy: 11	Primary anastomosis: 1		Sepsis: 1
			L. pneumonectomy: 1		Phonation problems: 1
			ML bilobectomy: 1		
Carretta (63)	30	Transcervical-transtracheal: 13	Primary suture: 29	3.3%	Pneumonia: 3
		Thoracotomy: 16	Primary anastomosis: 1		Phonation problems: 2
		Transcercical-transtracheal + thoracotomy:1			Tracheo-esophageal fistula recurrence: 1

*75% for patients treated with pneumonectomy.

R: right, L: left, ML: middle and lower, BPF: broncho-pleural fistula.

### Conservative management

Although Kiser et al. [12] showed that no treatment or conservative management is associated with higher rates of death, several studied have commented on the possibility for non-operative management in patients with postintubation tracheal lacerations [43,66-68]. The prerequisites for such management include: small lacerations (<2 cm), a tube’s cuff inflated distally to the site of the injury, adequate ventilation with PEEP and low tidal volumes, evacuation of the air form the pleural cavity once a chest tube is placed, not increasing subcutaneous emphysema and absence of signs of an ongoing infection. In a recent review Minambres et al. [11] reported 71 patients with postintubation tracheal rupture who underwent conservative treatment; the mortality among them was 14.5% compared to 30.4% for the remaining 111 patients having surgical repair. Recent studies also support the role of conservative treatment in selected patients with post-traumatic airway injuries as long as the abovementioned criteria are fulfilled and the patients present with a prohibitively high operative risk [69-71]. Currently the rate of conservative treatment in series of patients with TBI ranges from 33.3% to 94.4% [20]. A challenging scenario is that of an injury fulfilling the criteria for conservative treatment located to the distal intrathoracic trachea and carina. If selective bilateral intubation of the bronchi, as the one proposed by Conti et al. [43], cannot be performed the patient should be treated with surgery. Despite the favorable outcome reported so far, the patients should be under strict follow up and they should be referred to surgery if airway loss, inadequate ventilation or signs of infection and sepsis arise.

### Delayed presentation injuries

Approximately 5% to 80% of the tracheobronchial injuries can be missed during the first 24–48 hours after the trauma because of the nonspecific nature of the presenting symptoms and the fact that an airway injury may sometimes allow near normal ventilation [12,23,39,57,72]. With time the bronchus will be filled with fibro-granulation tissue and organizing hematoma. The natural history of these patients depends on whether an airway stenosis or a complete bronchial obstruction occurs. In the former recurrent pulmonary infections may lead to bronchiectasis and destruction of the lung parenchyma, while in the later the lung distally to a completely obstructed bronchus is filled with mucus and protected from infections [12]. Patients presenting with dyspnoea or recurrent pulmonary infections, a lobar or pulmonary opacification on chest x-rays and a history of major trauma should be evaluated via bronchoscopy; once a delayed TBI is diagnosed they should be referred for surgery. The choices for treatment include: 1). freshening of the edges of the injury and anastomosis in selected patients with early presentation [73], 2). bronchial sleeve resection [12] and 3). anatomic lung resection in those patients with severely distorted lung parenchyma [57]. It is essential that intra-operative evaluation of the viability of the pulmonary parenchyma is carried out, especially when the aspiration of the retained secretions can be followed by almost complete re-expansion of the atelectatic lung tissue. Protective, well vascularized, tissues should be used to buttress the anastomosis or the bronchial stump, mainly in patients with history of recurrent pulmonary infections. In a review of 27 patients undergoing elective surgery for delayed presentation of TBI between 1996 and 2007 Glazer et al. [74] reported that there was no perioperative death and the radiological and functional outcome was excellent in most patients.

## Conclusions

Although airway trauma is still highly lethal, good outcome can be expected after surgical treatment. However, basic prerequisites for that are an early diagnosis based on a high index of suspicion, thorough knowledge of the mechanisms of injury and early attainment of a safe and patent airway. Primary repair with intent to restore the integrity of the airway, minimize the loss of pulmonary parenchyma, maintain vocal function and avoid permanent tracheostomy should be the scope of every attempt to surgically treat an airway trauma. The surgical approach to the injury should be dictated by its location as found during bronchoscopy. Selected patients, mostly with iatrogenic injuries can be treated conservatively; strict follow up is necessary and surgery should be undertaken once airway loss, inadequate ventilation or infection/sepsis occurs. Delayed presentation airway injuries should be treated either with bronchial sleeve resection or formal anatomic lung resection; attention should be made intra-operatively for the evaluation of the viability of the pulmonary parenchyma distally to the site of the injury to avoid unnecessary major lung resection.

## Abbreviations

MPR: Multiplanar reformatting; TBI: Tracheobronchial injury; PEEP: Positive end-expiratory pressure.

## Competing interests

The authors declare that they have no competing interests.

## Authors’ contributions

All authors: 1) have made substantial contributions to conception and design, or acquisition of data, or analysis and interpretation of data; 2) have been involved in drafting the manuscript or revising it critically for important intellectual content; and 3) have given final approval of the version to be published.
